# Regulation of Melanocortin-4 Receptor Pharmacology by Two Isoforms of Melanocortin Receptor Accessory Protein 2 in Topmouth Culter (*Culter alburnus*)

**DOI:** 10.3389/fendo.2020.00538

**Published:** 2020-08-14

**Authors:** Min Tao, Ren-Lei Ji, Lu Huang, Si-Yu Fan, Ting Liu, Shao-Jun Liu, Ya-Xiong Tao

**Affiliations:** ^1^State Key Laboratory of Developmental Biology of Freshwater Fish, College of Life Sciences, Hunan Normal University, Changsha, China; ^2^Department of Anatomy, Physiology and Pharmacology, College of Veterinary Medicine, Auburn University, Auburn, AL, United States

**Keywords:** topmouth culter, melanocortin-4 receptor, melanocortin receptor accessory protein 2, signaling, constitutive activity

## Abstract

Melanocortin-4 receptor (MC4R) plays important roles in regulation of multiple physiological processes, and interaction of MC4R and melanocortin receptor accessory protein 2 (MRAP2) is suggested to play pivotal role in energy balance of vertebrates. Topmouth culter (*Culter alburnus*) is an economically important freshwater fish in China. Herein we cloned culter *mc4r, mrap2a*, and *mrap2b*. Culter *mc4r* consisted of a 981 bp open reading frame encoding a protein of 326 amino acids. qRT-PCR revealed that *mc4r, mrap2a*, and *mrap2b* were primarily expressed in the central nervous system. In the periphery, *mc4r* and *mrap2b* were expressed more widely in the male, while *mrap2a* was expressed more widely in the female. Culter MC4R could bind to four peptide agonists and increase intracellular cAMP production dose dependently. Culter MC4R was constitutively active in both cAMP and ERK1/2 pathways, which was differentially regulated by culter MRAP2a and MRAP2b. Culter MRAP2a significantly increased B_max_ and decreased agonist-stimulated cAMP, while MRAP2b increased cell surface and total expression but did not affect B_max_ and agonist-stimulated cAMP. These results will aid the investigation of the potential physiological processes that MC4R might be involved in topmouth culter.

## Introduction

The melanocortins are derived from tissue-specific post-translational processing of proopiomelanocortin (POMC), including α-, β-, γ-melanocyte-stimulating hormones (α-, β-, γ-MSH) and adrenocorticotropic hormone (ACTH) (1, 2). The physiological functions of these peptides are exerted by five melanocortin receptors (MC1R-MC5R), members of rhodopsin-like Family A G-protein-coupled receptors (GPCRs), including regulation of pigmentation, adrenal steroidogenesis, energy homeostasis, lipolysis, stress, cardiovascular, and sexual function (3–5). MC4R is highly expressed in the central nervous system and involved in regulating energy homeostasis via modulating both food intake and energy expenditure; it is also involved in regulating sexual function and reproduction by affecting the secretion of reproductive hormones [reviewed in (6, 7)]. Targeted deletion of *Mc4r* increases food intake and decreases energy expenditure, resulting in obesity in mice (8, 9). *MC4R* mutation is the leading cause of human monogenic obesity (10) [reviewed in (11, 12)].

The MC4R is primarily coupled to the stimulatory heterotrimeric G protein (Gs). MC4R activation leads to stimulation of adenylyl cyclase activity, which will increase the intracellular level of the second messenger cyclic adenosine monophosphate (cAMP) to trigger downstream signaling (13). MC4R activation also phosphorylates extracellular signal-regulated kinase 1 and 2 (ERK1/2) (14–17).

Melanocortin receptor accessory proteins (MRAPs, including MRAP1 and MRAP2), small single transmembrane-domain proteins, are identified as receptor-specific molecular chaperones, regulating MC2R trafficking, ligand binding and cAMP generation (18–21). MRAP2 is involved in modulating energy homeostasis due to its high expression in central nervous system. MRAP2 deceases agonist-stimulated signaling but does not affect basal signaling of human (h) MC4R (20), while MRAP2 in mice decreases the basal activity and increases maximal response (R_max_) (22). *MRAP2* mutations were shown to be potentially pathogenic for early-onset obesity (22, 23). Mice with whole body and brain-specific deletion of *Mrap2* also have early-onset severe obesity (22). These results suggest that MRAP2 is involved in MC4R signaling and regulating body weight in mammals (21, 22).

MC4R has also been characterized in teleosts (21, 24–32), and shown to act as a regulator in energy balance, sexual behavior, and reproduction (33–37). Non-mammalian MRAPs are different from those of mammals. Some fishes only have MRAP2, lacking MRAP1 (38, 39), and some have two MRAP2s (MRAP2a and MRAP2b) (21, 40). In zebrafish, two MRAP2s have different expression patterns at different stages, with MRAP2a expressed from embryos to adults, stimulating growth by blocking MC4R action in larvae, and MRAP2b mainly expressed in adults, enhancing MC4R response (21). *In vitro*, MRAP2a decreases the MC4R affinity for α-MSH, but MRAP2b increases ligand sensitivity of zebrafish MC4R (21). Hence, a better understanding of the vital functions of MC4R in modulating energy homeostasis and reproductive function and the interaction between MRAP2 and MC4R is important for feeding and artificial breeding of economical species.

The topmouth culter (*Culter alburnus*), which belongs to Cyprinidae, Cultrinae, Erythroculter, is an economically important freshwater fish widely distributed in large rivers, reservoirs, and lakes in China (41–44). With excellent taste, rapid growth, and strong performance in culture, this fish has been extensively cultured over the past few decades due to high market demand (45). In this study, we cloned culter *mc4r, mrap2a*, and *mrap2b*, and investigated the tissue distribution of these genes. We then studied the pharmacological properties of caMC4R and the effects of MRAP2a and MRAP2b on caMC4R.

## Materials and Methods

### Ligands and Plasmids

[Nle^4^, D-Phe^7^]-α-MSH (NDP-MSH) was purchased from Peptides International (Louisville, KY, USA), human α- and β-MSHs from Pi Proteomics (Huntsville, AL, USA), ACTH (1-24) from Phoenix Pharmaceuticals (Burlingame, CA, USA), and THIQ (N-[(3R)-1,2,3,4-Tetrahydroisoquinolinium-3-ylcarbonyl]-(1R)-1-(4-chlorobenzyl)-2-[4-cyclohexyl-4-(1H-1,2,4-triazol-1-ylmethyl)piperidin-1-yl]-2-oxoethylamine) (46) from Tocris Bioscience (Ellisville, MO, USA). We analyzed culter *pomc* from culter genome, and found that homology of culter α-MSH, ACTH (1-24) and β-MSH with human counterparts were 100, 87.5, and 57.1%, respectively (Supplementary Figure 1). [^125^I]-NDP-MSH and [^125^I]-cAMP were iodinated using chloramine T method (17, 47). The N-terminal c-myc-tagged wild-type hMC4R was subcloned into pcDNA3.1 vector as previously described (48). N-terminal c-myc-tagged caMC4R, N-terminal FLAG-tagged caMRAP2a, and N-terminal FLAG-tagged caMRAP2b were synthesized and subcloned into pcDNA3.1 vector by GenScript (Piscataway, NJ, USA) to obtain the expression plasmids.

### Gene Cloning and Sequence Alignment

Adult culter were collected from Engineering Research Center of Polyploid Fish Reproduction and Breeding of the Ministry of Education at Hunan Normal University. Fish were anesthetized before decapitation and tissues were excised and stored at −80°C. All experiments were approved by Animal Care Committee of Hunan Normal University and followed guidelines of the Administration of Affairs Concerning Experimental Animals of China.

Total RNA was purified using Trizol™ Reagent (Invitrogen, Carlsbad, CA, USA). PrimeScript RT reagent kit with gDNA Eraser (TaKaRa, Tokyo, Japan) was used for first-strand cDNA synthesis. Primer Premier 5.0 was used to design specific primers (Supplementary Table 1) to obtain partial cDNA fragment of the coding region and untranslated region (UTR) by PCR and touch-down PCR with TaKaRa LA Taq® (TaKaRa), respectively. PCR products were detected with 1.2% agarose gels. Target fragments were purified by SanPrep Column DNA Gel (Sangon Biotech, Shanghai, China), and then subcloned into the pMD18-T vector (TaKaRa), and sequenced (Sangon Biotech).

### Tissue Distribution of *mc4r, mrap2a*, and *mrap2b*

To analyze tissue distribution of these genes, the olfactory bulb, telencephalon, mesencephalon, medulla, cerebellum, pituitary, hypothalamus, liver, heart, head kidney, gonads, skin, kidney, muscle, spleen, and gill, were taken from three males and three females, respectively. qRT-PCR was carried out by a Prism 7,500 Sequence Detection System (ABI, Foster City, CA, USA) according to manufacturer's instructions. Primers were designed by AlleleID 6, and β*-actin* was used as the internal control (Supplementary Table 1). The reaction consisted of 5 μL SYBR green PCR Master Mix, 3 μL water, 1 μL cDNA sample, 0.5 μL QPCR-x-F, and 0.5 μL QPCR-x-R. The condition was: 50°C for 2 min, 95°C for 10 min, followed by 40 cycles at 95°C for 15 s and 61°C for 45 s. To ensure the accuracy, experimental samples were added to a 96-well plate repeated thrice. The 2^−ΔΔ*CT*^ method was used for analyzing the relative expression of the genes (49).

### Cell Culture and Transfection

Human embryonic kidney (HEK) 293T cells (ATCC, Manassas, VA, USA) were cultured at 37°C in a 5% CO_2_-humidified atmosphere. The medium contained Dulbecco's Modified Eagle's medium (DMEM) (Invitrogen, Carlsbad, CA, USA), 10% newborn calf serum, 10 mM HEPES, 50 μg/mL of gentamicin, 0.25 μg/mL of amphotericin B, 100 μg/mL of streptomycin and 100 IU/mL of penicillin. Cells were plated into 24-well or 6-well plates (Corning, NY, USA) pre-coated with 0.1% gelatin. At ~60–70% confluency, cells were transfected with plasmids by calcium phosphate precipitation method (50). Total DNA was normalized using empty vector pcDNA3.1.

### Flow Cytometry Assay

The influence of caMRAP2a or caMRAP2b on the cell surface and total expression levels of caMC4R was studied using flow cytometry as described earlier (31). Cells were plated into 6-well plates and transfected with caMC4R (N-terminal c-myc tag) and caMRAP2a or caMRAP2b plasmids in four ratios (1:0, 1:1, 1:3, and 1:5). The C6 Accuri Cytometer (Accuri Cytometers, Ann Arbor, MI, USA) was used for analysis. The empty vector (pcDNA3.1) fluorescence was used for background staining. The expression of the caMC4R was calculated as the percentage of 1:0 (caMC4R/caMRAP2a or caMC4R/caMRAP2b) group (51).

### Ligand Binding Assays

Binding assay was used to study the binding properties of caMC4R to different ligands as described previously (48, 52). Culter MC4R or hMC4R plasmid (0.25 μg/μL) were transfected into cells (6-well plate). The ligands and final concentrations were: NDP-MSH (from 10^−11^ to 10^−6^ M), β-MSH (from 10^−10^ to 10^−5^ M), α-MSH (from 10^−10^ to 10^−5^ M), ACTH(1-24) (from 10^−10^ to 10^−5^ M) or THIQ (from 10^−11^ to 10^−6^ M). To study the effects of caMRAP2a or caMRAP2b on the binding property of caMC4R, caMC4R (0.25 μg/μL) with caMRAP2a or caMRAP2b in two ratios (1:0 and 1:5) were transfected into cells (6-well plate), and two ligands, α-MSH (from 10^−10^ to 10^−5^ M) and ACTH(1-24) (from 10^−10^ to 10^−5^ M) were used.

### cAMP Assays

Radioimmunoassay for intracellular cAMP was performed as described previously (47, 48). The final concentration of ligands used for signaling assays were NDP-MSH (from 10^−11^ to 10^−6^ M), α-MSH (from 10^−10^ to 10^−5^ M), β-MSH (from 10^−10^ to 10^−5^ M), ACTH(1-24) (from 10^−10^ to 10^−5^ M), or THIQ (from 10^−10^ to 10^−5^ M).

To study the potential effect of caMRAP2a and caMRAP2b on caMC4R signaling, caMC4R (0.25 μg/μL) and caMRAP2a or caMRAP2b plasmids in two ratios (1:0 and 1:5) were co-transfected into cells (24-well plate), and two ligands, α-MSH (from 10^−10^ to 10^−5^ M) and ACTH(1-24) (from 10^−10^ to 10^−5^ M) were used. To study the dose-dependent effect of caMRAP2a or caMRAP2b on the R_max_ of cAMP levels to α-MSH stimulation, caMC4R (0.25 μg/μL) and caMRAP2a or caMRAP2b plasmids in four ratios (1:0, 1:1, 1:3 and 1:5) were co-transfected into cells (24-well plate). To explore the constitutive activity of Gs-cAMP, cells were transfected with caMC4R plasmid in different concentrations (0, 0.007, 0.015, 0.030, 0.060, 0.125, and 0.250 μg/μL) (6-well plate).

### ERK1/2 Phosphorylation Assay

To explore the constitutive pERK1/2 level, cells were transfected with caMC4R plasmid in different concentrations (0, 0.007, 0.015, 0.030, 0.060, 0.125, and 0.250 μg/μL). The phosphorylated ERK1/2 levels were detected as described previously (16, 17). α-MSH (10^−6^ M) was used for stimulation. Rabbit anti-pERK1/2 antibody (Cell Signaling Technology, Danvers, MA) and mouse anti-β-tubulin antibody (Developmental Studies Hybridoma Bank, University of Iowa, Iowa City, IA) were used in this study. ImageJ 1.44 software (National Institute of Health, Bethesda, MD) were used to quantify the films. The pERK1/2 levels were normalized as a ratio of pERK1/2 over β-tubulin in the same gel.

### Statistical Analysis

All data were shown as mean ± SEM. Prism 8.3 software (GraphPad, San Diego, CA, USA) was used to calculate parameters including maximal binding (B_max_), IC_50_, maximal response (R_max_), basal activity, and EC_50_. The significance of differences in ligand binding and signaling between caMC4R and hMC4R were determined by Student's *t*-test. Ligand binding, cAMP, flow cytometry parameters of caMC4R regulated by MRAP2s and ERK1/2 signaling were analyzed for significance of differences by one-way ANOVA. Statistical analysis was also performed with GraphPad Prism 8.3 software.

## Results

### Nucleotide and Deduced Amino Acid Sequences of caMC4R, caMRAP2a and caMRAP2b

**Figure 1:**
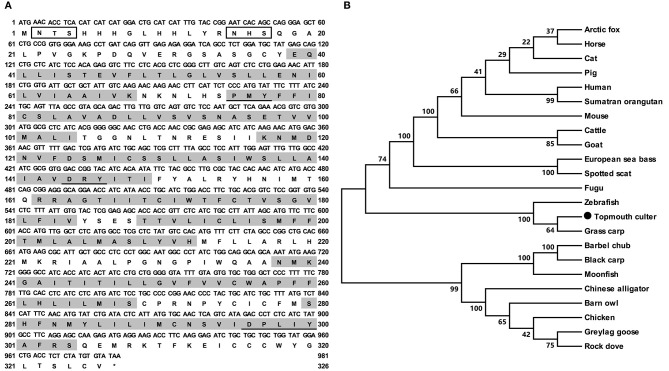
Nucleotide and deduced amino acid sequences and phylogenetic tree of caMC4R. **(A)** Nucleotide and deduced amino acid sequences of caMC4R. Positions of nucleotide and amino acid sequences are indicated on both sides. Shaded boxes denote putative TMD1-7. Potential phosphorylation sites are present in oval frame. Underline show PMY, DRY, DPxxY motifs. Open boxes denote the consensus sequence for *N*-linked glycosylation sites. Asterisk (*) indicates stop codon. **(B)** Phylogenetic tree of MC4R proteins. The tree was constructed by the neighbor-joining (NJ) method. Numbers at nodes indicate the bootstrap value, as percentages, obtained for 1,000 replicates. Black dot shows caMC4R. MC4Rs: *Culter alburnus* (topmouth culter, MT163518), *Scatophagus argus* (spotted scat, KU760724.1), *Dicentrarchus labrax* (European sea bass, CBN82190.1), *Lepisosteus oculatus* (spotted gar, XP_006634516.1), *Danio rerio* (zebrafish, NP_775385.1), *Vulpes lagopus* (arctic fox, ACN55093.1), *Ctenopharyngodon idella* (grass carp, AOZ60534.1), *Paralichthys olivaceus* (Japanese flounder, ADP09415.1), *Squaliobarbus curriculus* (barbel chub, ADV40875.1), *Takifugu rubripes* (fugu, NP_001027732.1), *Mylopharyngodon piceus* (black carp, ADV40871.1), *Tyto alba* (barn owl, ATN96237.1), *Columba livia* (rock dove, XP_021153678.1), *Bos taurus* (cattle, NP_776535.1), *Alligator sinensis* (Chinese alligator, XP_006025279.1), *Anser anser* (greylag goose, ABF19809.1), *Pongo abelii* (Sumatran orangutan, XP_002828309.1), *Equus caballus* (horse, XP_001489706.1), *Felis catus* (cat, XP_019670932.2), *Gallus gallus* (chicken, AEP17334.10), *Sus scrofa* (pig, ABD28176.1), *Mus musculus* (mouse, NP_058673.2), *Capra hircus* (goat, NP_001272520.1), and *Homo sapiens* (human, NP_005903.2).

The cloned topmouth culter *mc4r* had 981 bp open reading frame (ORF) that encoded a putative protein of 326 amino acids with 36.57 kDa molecular mass (Figure 1A). The culter MC4R had seven putative hydrophobic transmembrane domains (TMDs) with an extracellular N-terminus, three extracellular loops (ECLs), three intracellular loops (ICLs), and an intracellular C-terminus (Figure 1A and Supplementary Figure 2). The deduced amino acid sequence in the TMDs of caMC4R was significantly conserved with those of other species. The PMY, DRY, and DPxxY motifs were predicted at homologous positions with MC4Rs of other species (Supplementary Figure 2). Two potential *N*-linked glycosylation site (Asn^2^ and Asn^15^) in N-terminus, 15 cysteine residues and consensus sequence for protein kinase C phosphorylation (Thr^310^Phe^311^Lys^312^) in C-terminus were observed in the caMC4R primary structure (Supplementary Figure 2). By multiple sequence alignment analysis, we found that caMC4R shared high identities with other piscine MC4Rs, with 99.1% homology to grass carp, 98.5% to zebrafish, 86.9% to flounder, 87.7% to fugu, and 87.5% to sea bass, as well as to mammalian MC4Rs with 81.4% to human, 81.2% to mouse, and 82.0% to pig. Phylogenetic tree between caMC4R and other MC4Rs revealed that caMC4R was localized in a clade of grass carp and zebrafish (Figure 1B).

**Figure 2:**
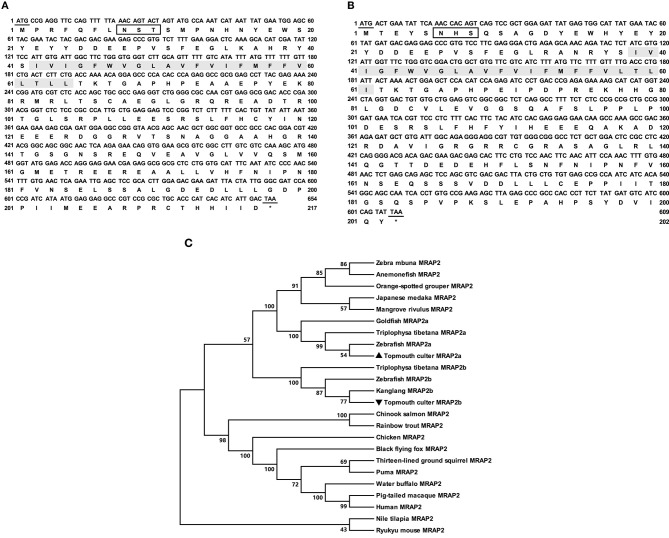
Nucleotide and deduced amino acid sequences and phylogenetic tree of caMRAP2a and caMRAP2b. **(A)** Nucleotide and deduced amino acid sequences of caMRAP2a. **(B)** Nucleotide and deduced amino acid sequences of caMRAP2b. The positions of nucleotide and amino acid sequences are indicated on both sides. Shaded box shows putative TMD. Open boxes denote putative *N*-linked glycosylation sites. Underline show initiation and stop codons. Asterisk (*) indicates stop codon. **(C)** Phylogenetic tree of MRAP2s. The tree was constructed by the NJ method. Numbers at nodes indicate the bootstrap value, as percentages, obtained for 1,000 replicates. Black triangle dot show caMRAP2a and caMRAP2b. MRAP2s: *Culter alburnus* (topmouth culter, MRAP2a: MT163516, and MRAP2b: MT163517), *Danio rerio* (zebrafish, MRAP2a: F8W4H9.1, and MRAP2b: F8W4H9.1), *Epinephelus coioides* (orange-spotted grouper, QED39647.1), *Oryzias latipes* (Japanese medaka, XP_023809099.1), *Maylandia zebra* (zebra mbuna, XP_004568825.1), *Amphiprion ocellaris* (anemonefish, XP_023122806.1), *Kryptolebias marmoratus* (mangrove rivulus, XP_017267334.1), *Oncorhynchus mykiss* (rainbow trout, XP_021467183.1), *Oreochromis niloticus* (Nile tilapia, XP_003458293.2), *Gallus gallus* (chicken, ALO81626.1), *Ictidomys tridecemlineatus* (thirteen-lined ground squirrel, XP_021581743.1), *Oncorhynchus tshawytscha* (Chinook salmon, XP_024278413.1), *Mus caroli* (Ryukyu mouse, XP_021029091.1), *Pteropus alecto* (black flying fox, XP_006926405.1), *Puma concolor* (puma, XP_025781535.1), *Homo sapiens* (human, AAH10003.2), *Bubalus bubalis* (water buffalo, XP_006054803.2), *Macaca nemestrina* (pig-tailed macaque, XP_011764298.1), *Triplophysa tibetana* (MRAP2a: KAA0703529.1 and MRAP2b: KAA0720858.1), *Anabarilius grahami* (Kanglang fish, MRAP2b ROJ29330.1), *Carassius auratus* (goldfish, MRAP2a: XP_026139519).

The cloned culter *mrap2a* had 654 bp ORF that encoded a putative protein of 217 amino acids with 24.37 kDa molecular mass (Figure 2A). The cloned culter *mrap2b* had 594 bp ORF that encoded a putative protein of 197 amino acids with 22.20 kDa molecular mass (Figure 2B). The culter MRAP2a and MRAP2b had similar features to other MRAP2s that contained a potential *N*-linked glycosylation site (Asn^8^ of caMRAP2a and Asn^6^ of caMRAP2b) in N-terminus, a single TMD, and a long C-terminal tail with many conserved residues (Supplementary Figure 3). In addition, a putative motif (LKAHKYS) was also found in caMRAP2a and caMRAP2b (Supplementary Figure 3), which is vital in the formation of antiparallel homodimers (53). Multiple sequence alignment showed that caMRAP2a shared high identity (93.6%) with zebrafish MRAP2a, and low identities (61.0%) with caMRAP2b and other piscine MRAP2, with 64.4% to rainbow trout MRAP2, 60.9% to tilapia MRAP2, as well as to mammalian MRAP2s with 61.8% to human MRAP2, 62.2% to mouse MRAP2 and 60.3% to goat MRAP2. Similar to caMRAP2a, caMRAP2b shared high identity with zebrafish MRAP2b with 80.9%, and low identities with other piscine MRAPs, with 61.1% to rainbow trout MRAP2, 59.0% to tilapia MRAP2, as well as to mammalian MRAP2s with 58.3% to human, 60.1% to mouse and 57.7% to goat. Phylogenetic tree analysis showed that caMRAP2a was clustered with teleost MRAP2a, nested with zebrafish MRAP2a, and MRAP2b was clustered with teleost MRAP2b, nested with kanglang fish MRAP2b (Figure 2C).

### Tissue Expression of Culter *mc4r, mrap2a*, and *mrap2b*

**Figure 3:**
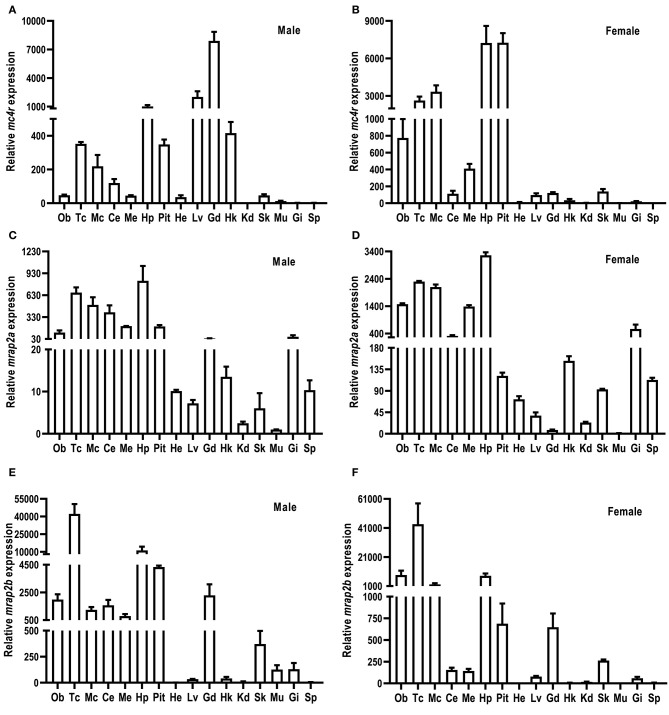
Expression of culter *mc4r* **(A,B)**, *mrap2a* **(C,D)** and *mrap2b* **(E,F)**. The mRNA levels of *mc4r, mrap2a*, and *mrap2b* were measured by qRT-PCR. The results were calculated as the ratio of β-actin. Each data point represented the mean ± SEM (*n* = 3). Ce, cerebellum; Ob, olfactory bulb; Mc, mesencephalon; Me, medulla; Tc, telencephalon; Pit, pituitary gland; Hp, hypothalamus; Lv, liver; He, heart; St, stomach; Int, intestine; Kd, kidney; Gd, gonad; Sk, skin; Sp, spleen; Hk, head kidney; Gi, gill; Mu, muscle.

The relative mRNA expression of culter *mc4r, mrap2a*, and *mrap2b* was analyzed by qRT-PCR (Figure 3). Sexual dimorphism was observed in *mc4r* expression (Figures 3A,B). Our results showed that in the male culter, *mc4r* was expressed more widely, in brain (telencephalon, mesencephalon, hypothalamus, medulla), pituitary gland, and the periphery (liver, testis, and head kidney) (Figure 3A). In the female culter, expression of *mc4r* was higher in mesencephalon, olfactory bulb, telencephalon, hypothalamus, medulla, and pituitary gland, but expressed at low levels in other peripheral tissues (Figure 3B).

Culter *mrap2a* was highly expressed in brain, and moderately expressed in testis and ovary. Culter *mrap2a* was expressed more widely in peripheral tissues in female than in male (Figures 3C,D). In the male, *mrap2a* was mainly expressed in testis and gill, and had a lower expression in other peripheral tissues studied (Figure 3C). In the female, *mrap2a* was present in liver, skin, kidney, gill, ovary, head kidney, and spleen (Figure 3D).

Similar to *mrap2a* distribution in the central nervous system, *mrap2b* was highly expressed in the olfactory bulb, telencephalon, cerebellum, mesencephalon, medulla, hypothalamus, and pituitary gland (Figures 3E,F). However, different from *marp2a* expression in peripheral tissues, *mrap2b* was present more widely in peripheral tissues of the male, including liver, head kidney, muscle, skin, testis, and gill (Figure 3E). In the female, *mrap2b* was primarily expressed in ovary and gill (Figure 3F).

### Ligand Binding Properties of caMC4R

**Figure 4:**
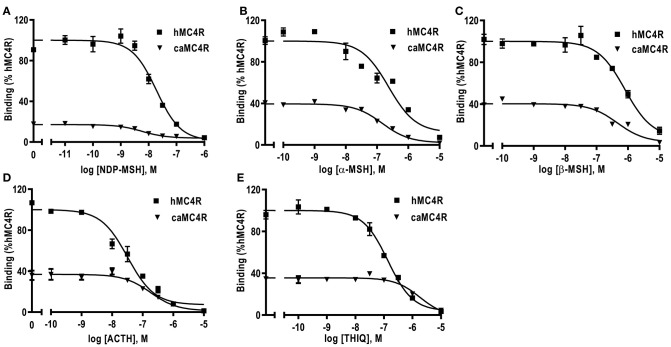
Ligand binding properties of caMC4R. HEK293T cells were transiently transfected with caMC4R plasmids (hMC4R was used as a control). Different concentrations of unlabeled NDP-MSH **(A)**, α-MSH **(B)**, β-MSH **(C)**, ACTH (1–24) **(D)**, and THIQ **(E)** was used to displace the binding of ^125^I-NDP-MSH. Results are expressed as % of hMC4R binding (in the absence of competitor) ± range from duplicate determinations within one experiment. All experiment was repeated at least three times independently.

Competitive ligand binding assays were performed to investigate the binding properties of caMC4R using hMC4R for comparison. Different concentrations of five unlabeled agonists (NDP-MSH, α-MSH, β-MSH, ACTH (1-24), and THIQ) were used as competitors with a fixed amount of ^125^I-NDP-MSH. The maximal binding values (B_max_) of caMC4R was 34.49 ± 3.43% of that of the hMC4R (Figure 4 and Table 1). Similar as the binding affinity order of hMC4R, caMC4R bound to superpotent agonist NDP-MSH with the highest affinity (IC_50_, 4.87 ± 1.80 nM), followed by ACTH (1–24) (123.03 ± 31.15 nM), α-MSH (126.33 ± 8.95 nM), and β-MSH (442.00 ± 65.43 nM) (Table 1). CaMC4R had a significantly higher affinity to β-MSH than hMC4R. THIQ was able to displace the ^125^I-NDP-MSH bound with caMC4R, although it had a lower affinity (1260.33 ± 272.61 nM) compared with that for hMC4R (164.63 ± 30.15 nM) (Figure 4 and Table 1).

**Table 1:** Ligand binding properties of caMC4R.

**MC4R**	**B_**max**_ %**	**NDP-MSH**	**α-MSH**	**β-MSH**	**ACTH**	**THIQ**
		**IC_**50**_ (nM)**	**IC_**50**_ (nM)**	**IC_**50**_ (nM)**	**IC_**50**_ (nM)**	**IC_**50**_ (nM)**
hMC4R	100	14.27 ± 2.98	280.47 ± 95.49	825.87 ± 66.17	61.96 ± 17.57	164.63 ± 30.15
caMC4R	34.49 ± 3.43^b^	4.87 ± 1.80	126.33 ± 8.95	442.00 ± 65.43^a^	123.03 ± 31.15	1260.33 ± 272.61

*Results are expressed as the mean ± SEM of at least three independent experiments. Human MC4R was used for comparison*.

a *Significantly different from the parameter of hMC4R, P < 0.05*.

b *Significantly different from the parameter of hMC4R, P < 0.001*.

### Signaling Properties of caMC4R

**Figure 5:**
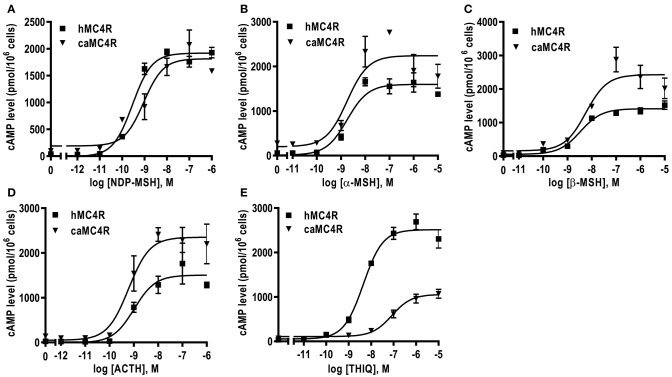
Signaling properties of caMC4R. HEK293T cells were transiently transfected with MC4R plasmids. Forty-eight hours after transfection, cells were stimulated with different concentrations of NDP-MSH **(A)**, α-MSH **(B)**, β-MSH **(C)**, ACTH (1–24) **(D)**, and THIQ **(E)**. Data are mean ± SEM from triplicate measurements within one experiment. All experiments were performed at least three times independently.

All agonists (NDP-MSH, α-MSH, β-MSH, ACTH (1-24) and THIQ) were able to stimulate caMC4R and dose-dependently increased intracellular cAMP levels (Figure 5 and Table 2). Similar maximal responses (R_max_) and EC_50_s to NDP-MSH, α-MSH, β-MSH, and ACTH (1-24) stimulation were observed between caMC4R and hMC4R. However, EC_50_ was significantly increased and R_max_ was significantly decreased when stimulated by THIQ (Figure 5 and Table 2).

**Table 2:** The signaling properties of hMC4R and caMC4R.

**MC4R**		**caMC4R**	**hMC4R**
Basal (%)		408.65 ± 103.30^a^	100
NDP-MSH	EC_50_ (nM)	0.44 ± 0.24	0.30 ± 0.05
	R_max_ (%)	84.48 ± 8.28	100
α-MSH	EC_50_ (nM)	1.23 ± 0.32	1.16 ± 0.32
	R_max_ (%)	166.21 ± 47.26	100
β-MSH	EC_50_ (nM)	5.76 ± 1.62	3.91 ± 1.09
	R_max_ (%)	167.74 ± 27.91	100
ACTH	EC_50_ (nM)	1.60 ± 0.76	1.16 ± 0.27
	R_max_ (%)	149.21 ± 26.94	100
THIQ	EC_50_ (nM)	75.71 ± 10.57^a^	5.70 ± 1.74
	R_max_ (%)	39.73 ± 1.37^b^	100

*Results are expressed as the mean ± SEM of at least three independent experiments*.

a *Significantly different from the parameter of hMC4R, P < 0.01*.

b *Significantly different from the parameter of hMC4R, P < 0.001*.

**Figure 6:**
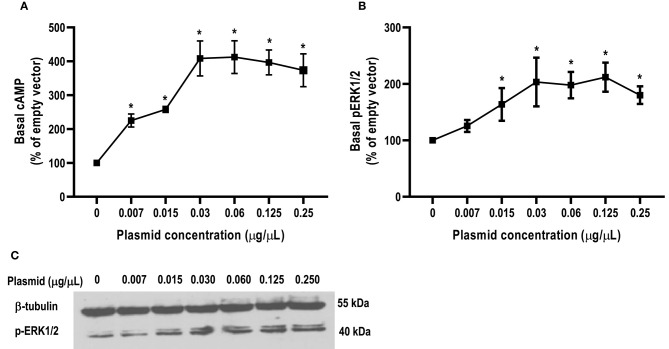
Constitutive activities of caMC4R in cAMP and ERK1/2 pathways. HEK293T cells were transfected with increasing concentrations of caMC4R plasmids. Cells transfected with empty pcDNA3.1 vector was considered as control group. **(A)** cAMP levels were were measured by RIA. The curve was made with data from three independent experiments. **(B,C)** The ERK1/2 phosphorylation levels were detected by western blot. Values are expressed as mean ± SEM (*n* = 3). Asterisk (*) showed significant difference of cAMP and pERK1/2 levels compared with control group (*P* < 0.05) (One-way ANOVA followed by Tukey-test).

In this study, we found that the basal signaling of caMC4R was 4.08 times that of hMC4R (Table 2), indicating that caMC4R might be constitutively active. Different concentrations of caMC4R plasmid were transfected into cells and basal intracellular cAMP levels measured. We found that a low amount of caMC4R plasmid (0.007 μg/μL) transfected resulted in high-level cAMP production (Figure 6A). Similar results were found in basal ERK1/2 phosphorylation, starting with a transfection of 0.015 μg/μL caMC4R that significantly increased basal pERK1/2 level (Figures 6B,C). Therefore, our data indicated that caMC4R could be constitutively active in both cAMP and ERK1/2 pathways.

### Modulation of caMC4R Expression and Pharmacology by caMRAP2s

**Figure 7:**
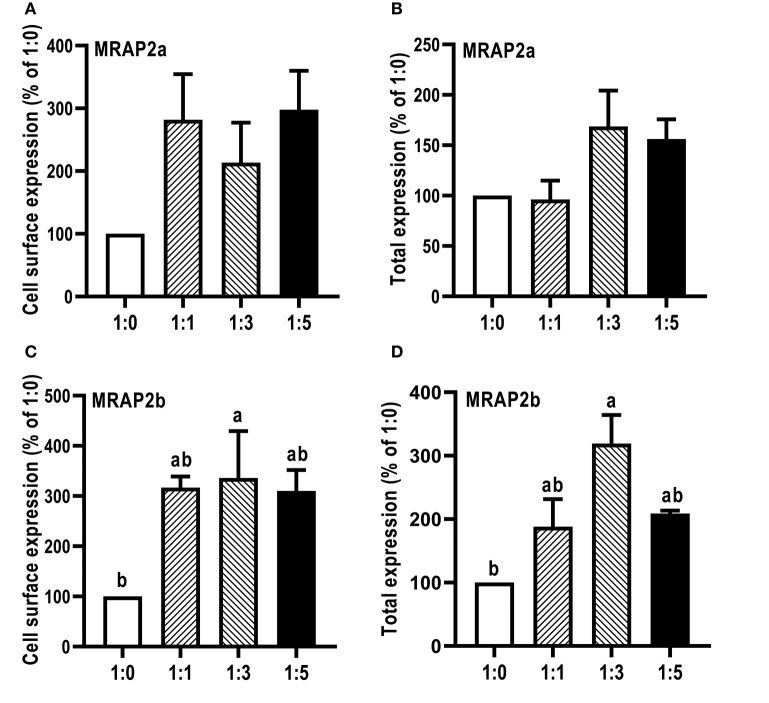
Modulation of caMC4R expression by caMRAP2a or caMRAP2b. Cell surface **(A,C)** and total expression **(B,D)** of caMC4R was measured by flow cytometry. HEK293T cells were cotransfected with different ratios of caMC4R/caMRAP2a or caMC4R/caMRAP2b (1:0, 1:1, 1:3, and 1:5). The results were calculated as % of 1:0 group after correction of the background staining (fluorescence in cells transfected with empty vector, pcDNA3.1). Each data point represented the mean ± SEM (*n* = 3–4). Different letters indicated significant difference (*P* < 0.05) (One-way ANOVA followed by Tukey-test).

The influence of caMRAP2a and caMRAP2b on the cell surface and total expression levels of caMC4R was performed by flow cytometry (Figure 7). The results demonstrated that caMRAP2a increased the cell surface and total expression of caMC4R, and there were no significant differences among groups (Figures 7A,B). Culter MRAP2b significantly increased cell surface and total expression of caMC4R in 1:3 group compared with the 1:0 group (Figures 7C,D).

**Figure 8:**
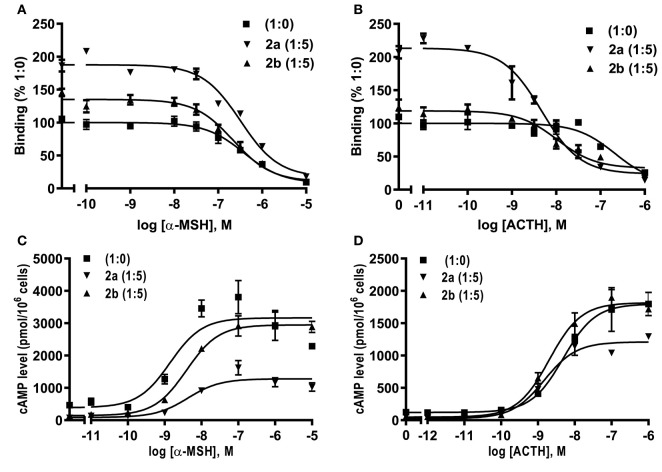
Modulation of caMC4R pharmacology by caMRAP2a or caMRAP2b. Binding **(A,B)** and signaling properties **(C,D)** of caMC4R to α-MSH or ACTH (1–24) upon co-expression of caMRAP2a or caMRAP2b were measured. HEK293T cells were co-transfected with caMC4R/caMRAP2a or caMC4R/caMRAP2b in two different ratios (1:0, and 1:5). Binding and signaling properties were calculated as described in detail in Figures 4, 5, respectively.

Competitive ligand binding assays with ACTH(1-24) and α-MSH showed that caMRAP2a significantly increased the B_max_ of caMC4R in the 1:5 group, while caMRAP2b did not affect the B_max_ (Figures 8A,B and Table 3). CaMRAP2a and caMRAP2b significantly increased affinity of caMC4R to ACTH(1-24) but did not affect the IC_50_s of caMC4R to α-MSH (Table 3).

**Table 3:** The effect of caMRAP2a or caMRAP2b on ligand binding properties of caMC4R.

**caMC4R/caMRAP2a or**	**B_**max**_**	**α-MSH**	**ACTH**
**caMRAP2b**			
		**IC_**50**_ (nM)**	**IC_**50**_ (nM)**
caMC4R (1:0)	100	229.00 ± 79.70	235.13 ± 16.70
caMC4R/caMRAP2a (1:5)	170.74 ± 13.08^a^	233.07 ± 44.80	6.34 ± 1.07^a^
caMC4R/caMRAP2b (1:5)	123.58 ± 9.86	153.47 ± 33.83	10.43 ± 1.12^a^

*Results are expressed as the mean ± SEM of at least three independent experiments*.

a *Significantly different from the parameter of 1:0, P < 0.001*.

To study the effect of caMRAP2a or caMRAP2b on the cAMP signaling of caMC4R, cells were co-transfected with caMC4R/caMRAP2a or caMC4R/caMRAP2b in two different ratios (1:0 and 1:5) and ACTH (1-24) and α-MSH were used as agonists. The results showed that caMRAP2a or caMRAP2b had no effect on EC_50_s; caMRAP2a significantly decreased the R_max_, but caMRAP2b did not affect the R_max_ (Figures 8C,D and Table 4).

**Table 4:** The effect of caMRAP2a or caMRAP2b on cAMP signaling properties of caMC4R.

**caMC4R/caMRAP2a or**	**α-MSH**		**ACTH**	
**caMRAP2b**			
	**EC_**50**_ (nM)**	**R_**max**_**	**EC_**50**_ (nM)**	**R_**max**_**
caMC4R (1:0)	1.53 ± 0.15	100	3.63 ± 1.21	100
caMC4R/caMRAP2a (1:5)	5.93 ± 1.73	47.60 ± 7.94^a^	1.29 ± 0.34	60.18 ± 3.62^a^
caMC4R/caMRAP2b (1:5)	4.76 ± 1.13	85.21 ± 5.27	1.75 ± 0.15	98.86 ± 4.02

*Results are expressed as the mean ± SEM of at least three independent experiments*.

a *Significantly different from the parameter of 1:0, P < 0.001*.

**Figure 9:**
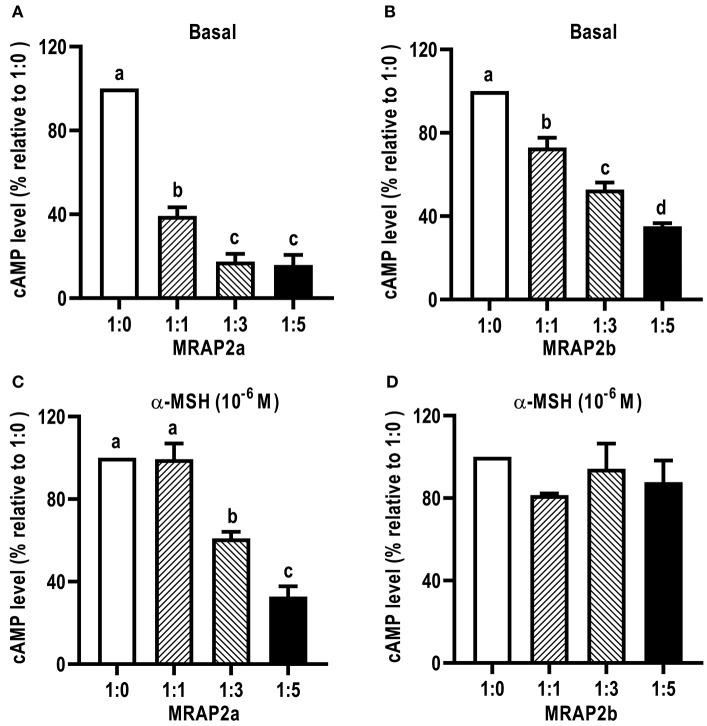
The effects of caMRAP2a or caMRAP2b on caMC4R signaling. HEK293T cells were co-transfected with different ratios of caMC4R/caMRAP2a or caMC4R/caMRAP2b (1:0, 1:1, 1:3, and 1:5) and cAMP levels under basal **(A,B)** or stimulated [with 10^−6^ M α-MSH, **(C,D)**] conditions were measured. Data are expressed as % of 1:0 group. Values are expressed as mean ± SEM (*n* = 3). Different letters indicate significant difference (one-way ANOVA followed by Tukey-test).

We further investigated the dose-dependent effects of caMRAP2a or caMRAP2b on basal and maximal signaling to α-MSH stimulation (Figure 9). Cells were co-transfected with four ratios of caMC4R/caMRAP2a or caMC4R/caMRAP2b (1:0, 1:1, 1:3, and 1:5). We found that the basal cAMP production of caMC4R were dose-dependently decreased by both caMRAP2a and caMRAP2b (Figures 9A,B). In addition, maximal cAMP levels of caMC4R activated by 10^−6^ M α-MSH were also dose-dependently decreased by caMRAP2a in 1:3 and 1:5 groups but not caMRAP2b (Figures 9C,D).

## Discussion

In the present study, we demonstrated that cloned culter *mc4r* was predicted to encode a protein of 326 amino acids with similar structural characteristics as MC4Rs of other species (Figure 1A and Supplementary Figure 2), including other teleost MC4Rs (27–31, 37). Phylogenetic analysis revealed that caMC4R clustered with teleost MC4Rs (Figure 1B).

The distribution of MC4R in lower vertebrates is much wider than in mammals. We observed that culter *mc4r* was highly expressed in the central nervous system (Figure 3), consistent with it being a critical regulator of energy homeostasis (7, 24, 28, 30). In addition to brain, culter *mc4r* was present more widely in the periphery in the male, especially in the testis (but not in the ovary) (Figures 3A,B). These data suggest that teleost MC4Rs might play an important role in regulating reproductive function (28, 30, 37). Several studies have investigated the roles of teleost MC4R in regulating reproductive function (33, 35–37, 54, 55).

In the present study, we also cloned culter *mrap2a* and *mrap2b*, and showed that similar to MRAP2s of other species, culter MRAP2a and MRAP2b had one potential *N*-linked glycosylation site in the N-terminal domain and a single highly conserved TMD (Figure 2 and Supplementary Figure 3). Based on culter genomic data (43), we did not identify MRAP1 in topmouth culter, consistent with the hypothesis that MRAP1 is lost in lobe-finned fish, amphibians, and reptiles (39, 56). The tissue expression data showed that *mrap2a* and *mrap2b* were highly expressed in the central nervous system (Figures 3C–F), similar to the expression of *mc4r*, indicates that caMRAP2a or caMRAP2b might modulate MC4R signaling in the central nervous system. In the periphery, *mrap2a* was expressed more widely in the female than in the male, while *mrap2b* was expressed more widely in the male (Figures 3C–F). Especially, *mrap2b* was highly expressed in the testis and ovary, while *mrap2a* had lower expression in the testis and ovary (Figures 3C–F). These data suggested that caMRAP2a and caMRAP2b might have differential roles in modulating MC4R signaling in the periphery, especially in regulating gonadal function.

We also explored the pharmacology of the cloned caMC4R with binding and signaling assays. Our results showed that NDP-MSH bound to caMC4R with the highest affinity (IC_50_ of 4.87 nM) and activated caMC4R with the highest potency (EC_50_ was 0.44 nM) (Tables 1, 2). Lower binding capacity was observed in caMC4R (about 35% of that of hMC4R) (Figure 4 and Table 1), consistent with previous studies in spotted scat (27), grass carp (28), swamp eel (29), sea bass (31), and orange-spotted grouper (30). In culter, ACTH had higher affinity and was more efficacious than α-MSH for caMC4R, consistent with the suggestion that ACTH may be the original ligand for the MCRs (57).

In cAMP signaling assay, α-MSH, β-MSH and ACTH (1-24) stimulated caMC4R and hMC4R with similar potencies (Figure 5 and Table 2). THIQ, a small molecule agonist, displaced ^125^I-NDP-MSH from caMC4R with a lower affinity than hMC4R (Table 1), activated caMC4R and initiated cAMP accumulation with an EC_50_ of 75.71 nM (~15-fold higher than that of hMC4R) (Table 2). These data suggested that THIQ was not an allosteric agonist at caMC4R, different from our previous studies in grass carp and swamp eel (28, 29).

The hMC4R has been shown to have constitutive activity in Gs-cAMP signaling (58), and N-terminus is an important modulator in regulating constitutive activities in hMC4R (59). Mutations leading to decreased constitutive activity are associated with obesity pathogenesis (6, 59, 60). Compared to hMC4R, teleost MC4Rs showed much higher constitutive activity in cAMP signaling (27–31). Our present study also showed that caMC4R significantly increased basal activities in Gs-cAMP and ERK1/2 signaling (Figure 6). The potential relevance of constitutive activity in teleost MC4Rs remains to be studied more extensively.

We further investigated whether MRAP2a and MRAP2b could modulate the trafficking, ligand binding and signaling of caMC4R. CaMRAP2a and caMRAP2b both increased the cell surface expression of caMC4R (Figure 7). In zebrafish, MRAP2b dose-dependently increases the cell surface expression of MC4R, while MRAP2a has no effect on the cell surface expression of MC4R (21). Mouse MRAP1 and MRAP2 decrease the cell surface expression of MC4R (20). In chicken, MRAP and MRAP2 have no significant effect the cell surface expression of MC4R (61). In tilapia, MRAP2 dose-dependently decreases the cell surface expression of MC4R (32). MRAP2 also decreases the cell surface expression of MCRa and MCRb in sea lamprey, which has only two MCRs (62). Therefore, the effect of MRAP2 on cell surface and total expression of the MC4R varies in different species. CaMRAP2a increased the B_max_ of caMC4R but caMRAP2b did not affect the B_max_ (Table 3). caMRAP2a and caMRAP2b significantly increased affinity of caMC4R to ACTH(1-24) but did not affect the IC_50_ of caMC4R to α-MSH (Table 3).

Remarkably, culter MRAP2a and MRAP2b also inhibited the constitutive activity of caMC4R (Figure 9). Furthermore, caMRAP2a significantly decreased the R_max_ but caMRAP2b had no effect on the R_max_ (Table 4). MRAP2 decreases the constitutive activity of MC4R and the R_max_ of MC4R in tilapia and grouper (30, 32). In zebrafish, MRAP2a suppresses the constitutive activity of MC4R, reduces the R_max_, while increases α-MSH potency and MRAP2b suppresses the constitutive activity of MC4R and increases the R_max_ (21). In sea lamprey, MRAP2 increases agonist-stimulated signaling of MCRa and MCRb (62). In chicken, MRAP and MRAP2 decreases the basal activity and increases sensitivity to ACTH (61). In mouse, MRAP1 and MRAP2 decreases agonist-stimulated cAMP production (20). Therefore, different effects of MRAPs on MC4R basal and agonist-stimulated signaling are observed in different species.

One shortcoming of this study is that we used human ACTH and β-MSH for the experiments. α-MSH has been shown to be fully conserved in all species with POMC gene studied so far, including culter investigated here. For ACTH(1-24), there were 3 amino acids different between human and culter sequences, but two of these changes were very conservative (Supplementary Figure 1). Therefore, we deduce that culter ACTH(1-24) would likely behave similarly as human ACTH(1-24) that we used in the experiments. There was only 57.1% homology between human and culter β-MSHs (Supplementary Figure 1). Therefore, we need to interpret the data obtained for β-MSH with caution. In future studies, we need to identify the molecular forms of the endogenous MSHs produced and the modifications (for example, acetylated or des-acetylated) and synthesize these peptides for functional characterization experiments.

In summary, we cloned and analyzed the expression patterns of *mc4r, mrap2a*, and *mrap2b* from topmouth culter. All three genes were mainly present in the central nervous system, but differential expression was observed in the periphery. Culter MC4R had high constitutive activities and similar potencies to several agonists as hMC4R. Culter MRAP2a significantly increased the B_max_ and decreased agonist-stimulated cAMP, whereas culter MRAP2b increased the cell surface and total expression but did not affect B_max_ and agonist-stimulated cAMP. Therefore, these data suggested that caMRAP2a and caMRAP2b had differential effects on the expression, binding, and signaling of caMC4R. These findings lay the foundation for future physiological studies on the functions of culter MC4R that might provide new strategies to improve growth and reproduction in culter culture.

## Data Availability Statement

The raw data supporting the conclusions of this article will be made available by the authors, without undue reservation.

## Ethics Statement

The animal study was reviewed and approved by Animal Care Committee of Hunan Normal University.

## Author Contributions

MT: conceptualization, investigation, writing original draft, and funding acquisition. R-LJ: conceptualization, investigation, and writing original draft. LH, S-YF, and TL: investigation. S-JL and Y-XT: conceptualization, writing–review and editing, supervision, project administration, and funding acquisition. All authors contributed to the article and approved the submitted version.

## Conflict of Interest

The authors declare that the research was conducted in the absence of any commercial or financial relationships that could be construed as a potential conflict of interest.
