# Supplementary material for: Regulation of Melanocortin-4 Receptor Pharmacology by Two Isoforms of Melanocortin Receptor Accessory Protein 2 in Topmouth Culter (Culter alburnus)
Source: Front Endocrinol (Lausanne). 2020 Aug 14;11:538. doi: 10.3389/fendo.2020.00538 (PMC7456811; doi:10.3389/fendo.2020.00538)
Supplement: Supplementary file 1 [file Presentation_1.PPTX]

## Slide 1
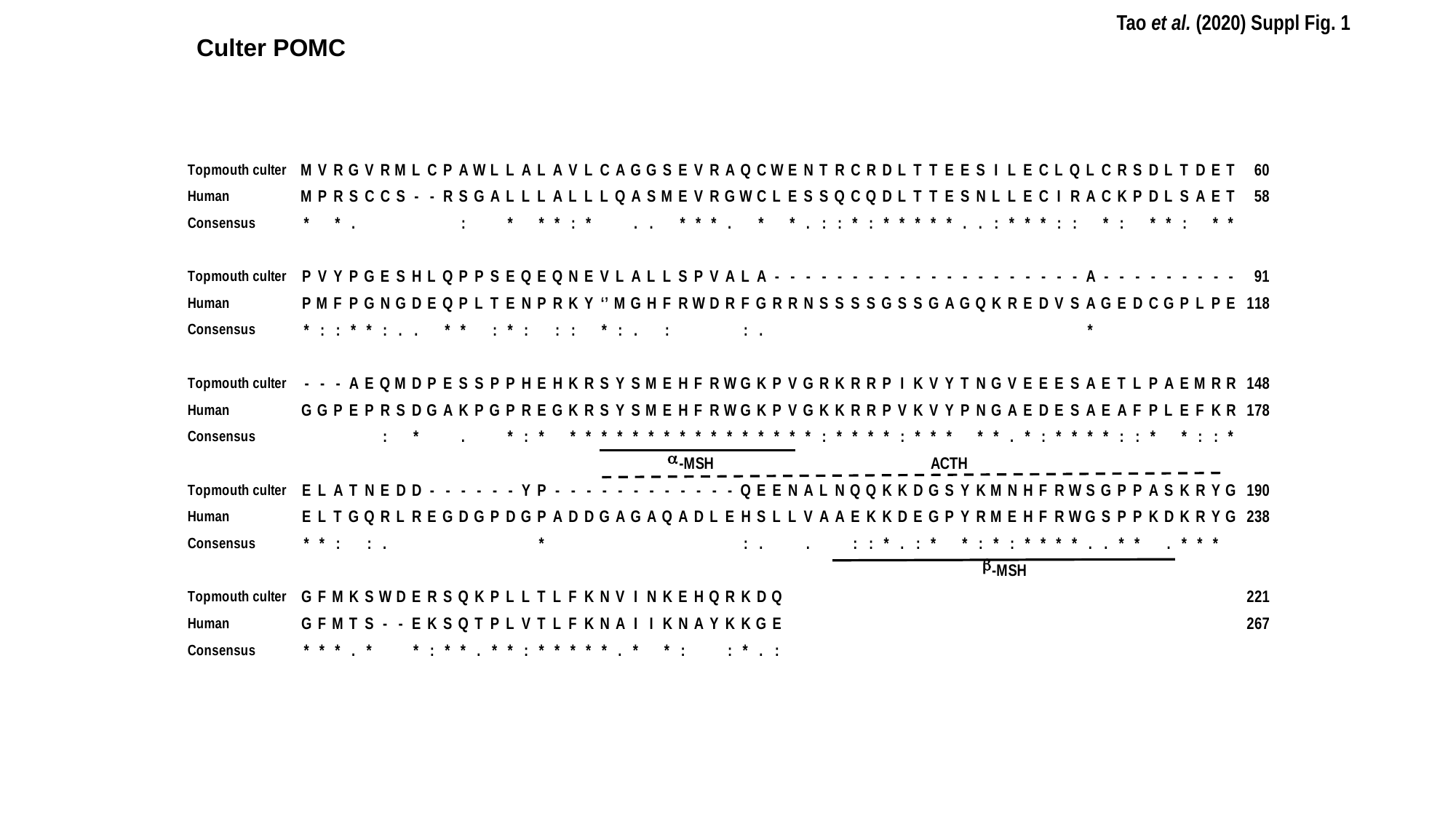

Tao et al. (2020) Suppl Fig. 1
Culter POMC

## Slide 2
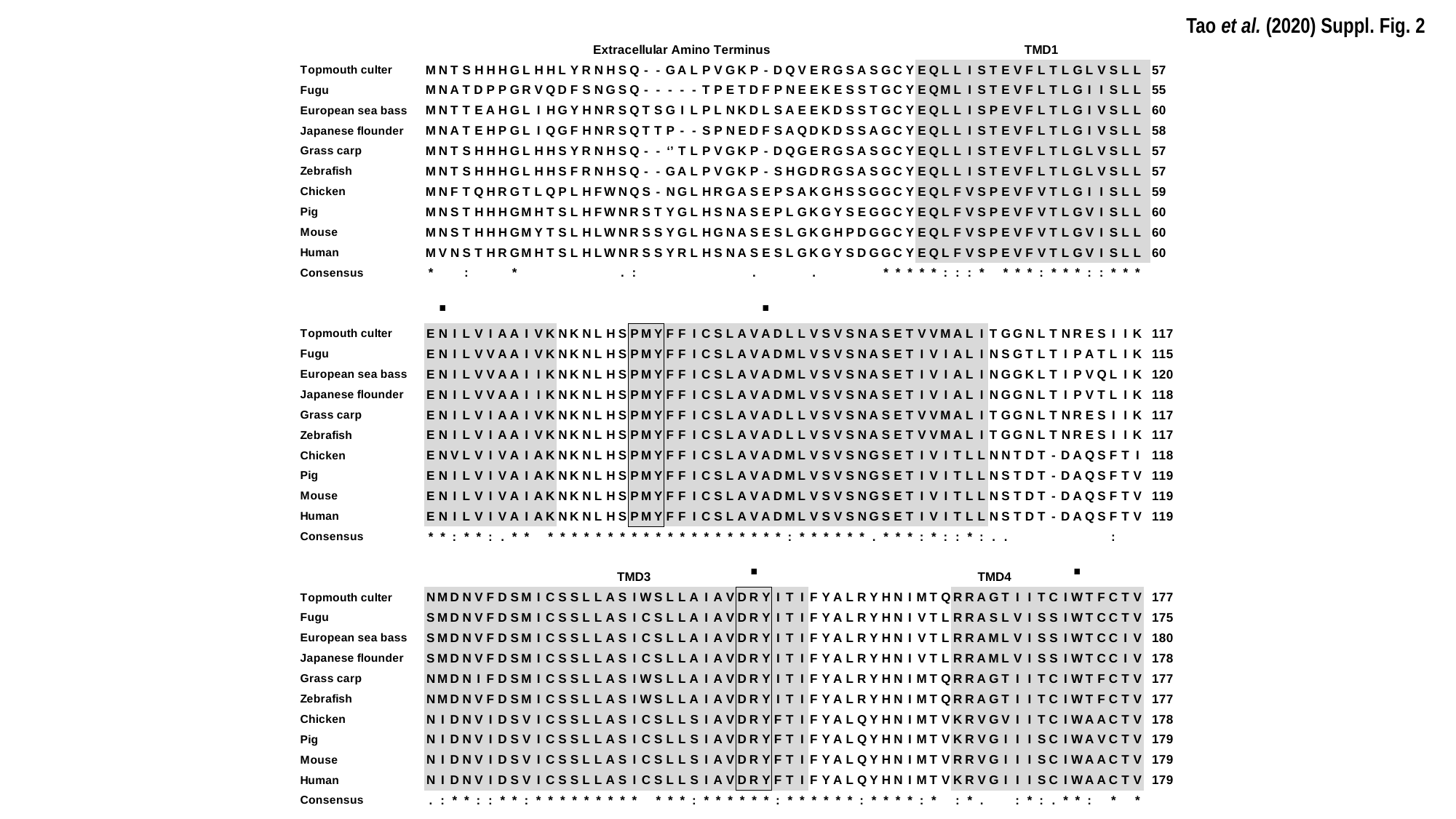

Tao et al. (2020) Suppl. Fig. 2

## Slide 3
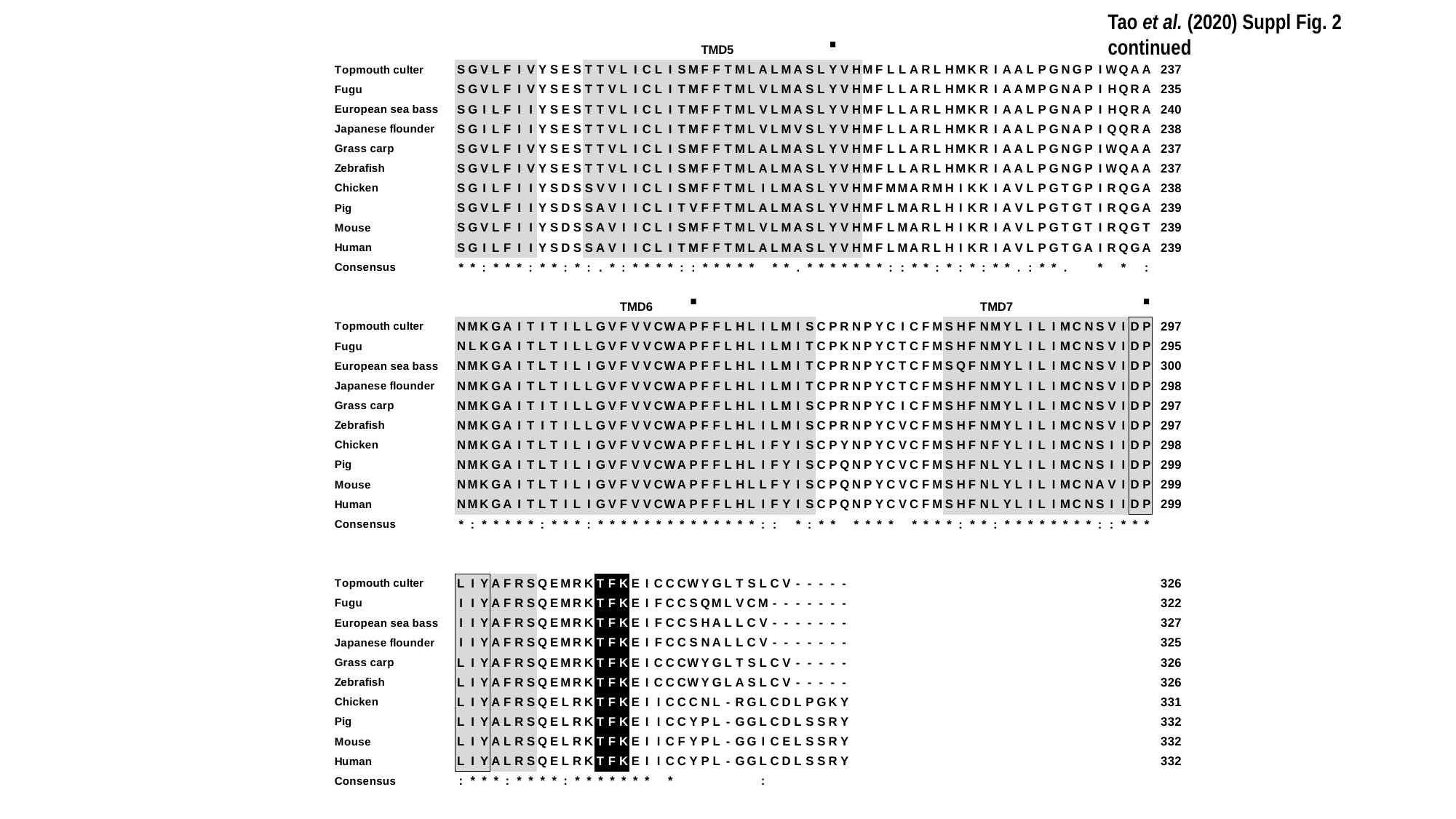

Tao et al. (2020) Suppl Fig. 2 continued

## Slide 4
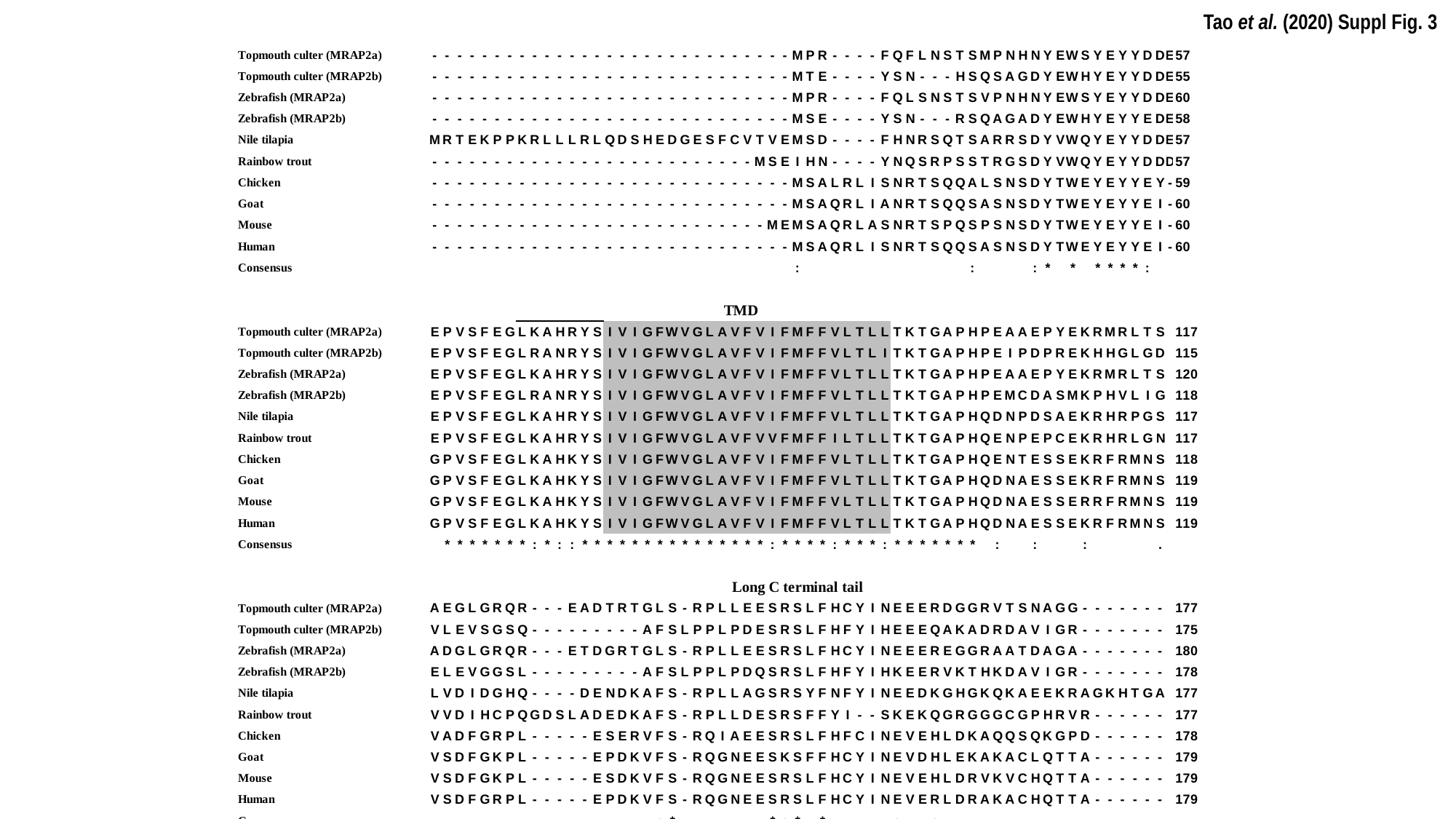

Tao et al. (2020) Suppl Fig. 3

## Slide 5
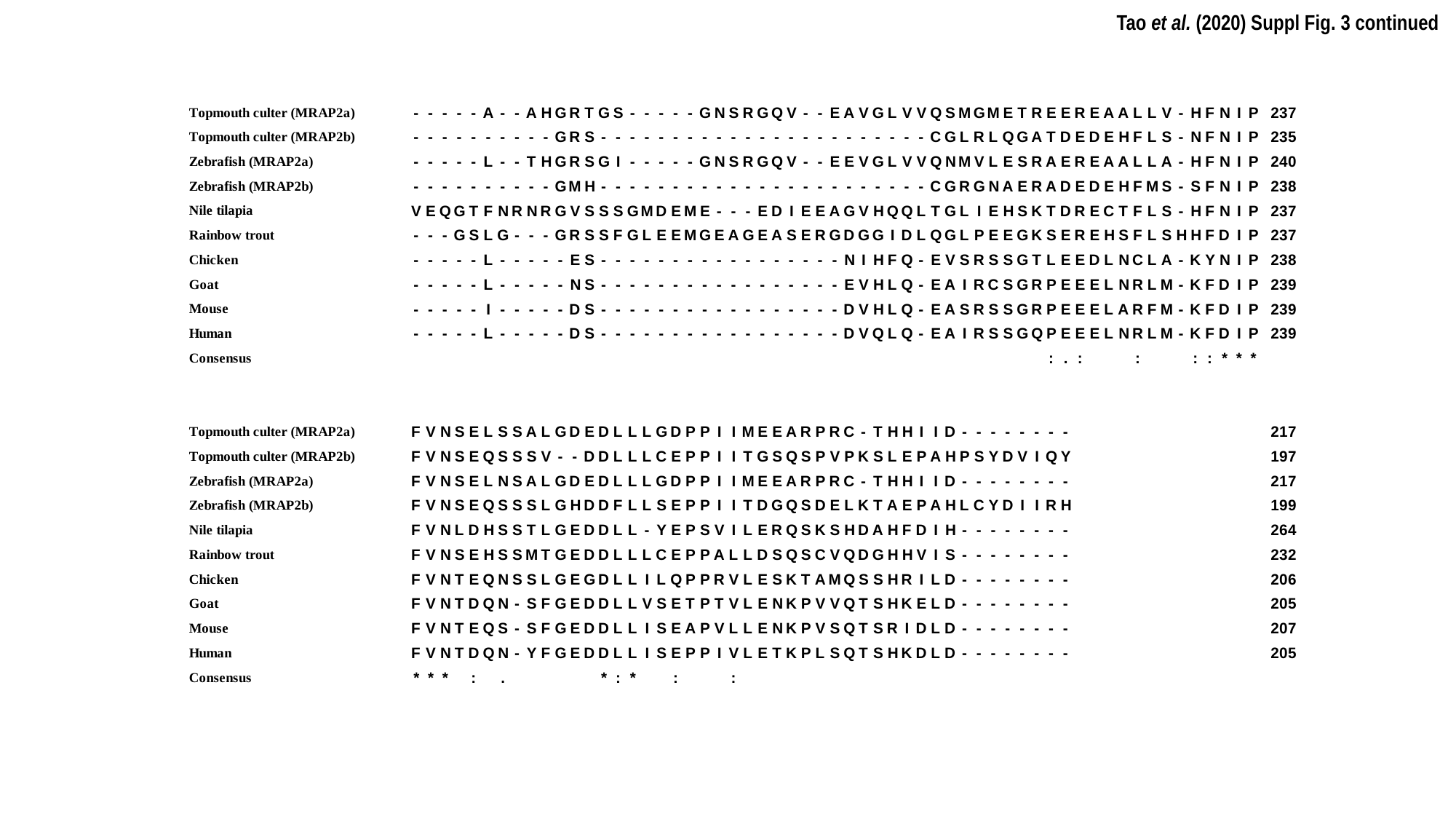

Tao et al. (2020) Suppl Fig. 3 continued
