# Supplementary material for: Regulation of Melanocortin-4 Receptor Pharmacology by Two Isoforms of Melanocortin Receptor Accessory Protein 2 in Topmouth Culter (Culter alburnus)
Source: Front Endocrinol (Lausanne). 2020 Aug 14;11:538. doi: 10.3389/fendo.2020.00538 (PMC7456811; doi:10.3389/fendo.2020.00538)
Supplement: Supplementary file 2 [file Table_1.DOCX]

**Supplementary Table 1 Primers used for cDNA cloning and qPCR.**

| Primer names | Primer sequence (5′–3′) | Purpose |
| --- | --- | --- |
| MC4R-F | GAACACCTCACATCATCA | PCR |
| MC4R-R | AGCACACTACAAATACCC | PCR |
| MRAP2a-F | AGCCCGTGTCTTTTGAA | PCR |
| MRAP2a-R | GATGTGATGGGTGCAGC | PCR |
| MRAP2b-F | GCTGGAGATTATGAGTGGC | PCR |
| MRAP2b-R | TGGAATGTTGAAGTTGGAC | PCR |
| MC4R-3′-1 | CGTTCTCATCTGCCTTATTAGC | 3′ RACE |
| MC4R-3′-2 | TATCCTGCTGGGGGTATTTGTA | 3′ RACE |
| MRAP2a-3′-1 | AGAAACGGATGCGTCTCACCAGC | 3′ RACE |
| MRAP2a-3′-2 | GGAGACCAGGGAGGAACGAGAGG | 3′ RACE |
| MRAP2b-3′-1 | CTCTTTCACTTCTACATCCACG | 3′ RACE |
| MRAP2b-3′-2 | GACGAGCACTTCCTGTCCAACT | 3′ RACE |
| MC4R-5′-1 | CCAGGAGACTGACAAGCCCGAGCG | 5′ RACE |
| MC4R-5′-2 | TCAGGCTTTCCCACCGGCAGAG | 5′ RACE |
| MRAP2a-5′-1 | ACGCATCCGTTTCTCATAAGGCTC | 5′ RACE |
| MRAP2a-5′-2 | TGAGTCCTTCAAAAGACACGGGCT | 5′ RACE |
| MRAP2b-5′-1 | CCTCGTGGATGTAGAAGTGAAA | 5′ RACE |
| MRAP2b-5′-2 | GACCTCCAGCACACAGTCACCT | 5′ RACE |
| MC4R-qF | AGCACCACCGTTCTCATCTG | qRT-PCR |
| MC4R-qR | CCAGGGAGGGCAGCAATG | qRT-PCR |
| MRAP2a-qF | GGCAACTCAAGAGGACAG | qRT-PCR |
| MRAP2a-qR | CCAATAGTAAATCTTCGTCTCC | qRT-PCR |
| MRAP2b-qF | CCTCTTTCACTTCTACATCCAC | qRT-PCR |
| MRAP2b-qR | GCTCGTCTTCGTCTGTCG | qRT-PCR |
| Actin-qF | TCACACCTTCTACAACGAGCTGCG | qRT-PCR |
| Actin-qR | GAAGCTGTAGCCTCTCTCGGTCAG | qRT-PCR |
